# Shared and Unique Evolutionary Trajectories to Ciprofloxacin Resistance in Gram-Negative Bacterial Pathogens

**DOI:** 10.1128/mBio.00987-21

**Published:** 2021-06-22

**Authors:** Jaime E. Zlamal, Semen A. Leyn, Mallika Iyer, Marinela L. Elane, Nicholas A. Wong, James W. Wamsley, Maarten Vercruysse, Fernando Garcia-Alcalde, Andrei L. Osterman

**Affiliations:** a Sanford Burnham Prebys Medical Discovery Institute, La Jolla, California, USA; b Roche Pharma Research and Early Development, Immunology, Inflammation, and Infectious Diseases, Basel, Switzerland; The Ohio State University

**Keywords:** antibiotic resistance, ciprofloxacin, experimental evolution, Gram-negative bacteria, morbidostat

## Abstract

Resistance to the broad-spectrum antibiotic ciprofloxacin is detected at high rates for a wide range of bacterial pathogens. To investigate the dynamics of ciprofloxacin resistance development, we applied a comparative resistomics workflow for three clinically relevant species of Gram-negative bacteria: Escherichia coli, Acinetobacter baumannii, and Pseudomonas aeruginosa. We combined experimental evolution in a morbidostat with deep sequencing of evolving bacterial populations in time series to reveal both shared and unique aspects of evolutionary trajectories. Representative clone characterization by sequencing and MIC measurements enabled direct assessment of the impact of mutations on the extent of acquired drug resistance. In all three species, we observed a two-stage evolution: (i) early ciprofloxacin resistance reaching 4- to 16-fold the MIC for the wild type, commonly as a result of single mutations in DNA gyrase target genes (*gyrA* or *gyrB*), and (ii) additional genetic alterations affecting the transcriptional control of the drug efflux machinery or secondary target genes (DNA topoisomerase *parC* or *parE*).

## INTRODUCTION

Increasing antibiotic resistance is a premier threat to modern medicine. This danger necessitates expanded research into the mechanisms by which organisms gain resistance and continued development of new drugs to replace those becoming ineffective. A fluoroquinolone antibiotic, ciprofloxacin (CIP), was introduced for medical use in 1987 and boasted a wider spectrum of efficacy than those of quinolones (1). Ciprofloxacin is commonly prescribed as a front-line treatment against a broad range of bacterial infections (2, 3). Quinolone antibiotics target bacterial DNA gyrase (GyrA/GyrB) and topoisomerase IV (ParC/ParE), enzymes which control DNA supercoiling during replication and transcription. By binding to these enzymes when they are complexed with DNA, CIP inhibits the repair of DNA breaks and causes irreversible damage to the genome (4–8).

A weak-to-moderate resistance to CIP can occur through a single missense mutation in one of the target genes, yielding resistant variants, even when selected at drug levels substantially below the MIC. Thus, the most common CIP-resistant variant of Escherichia coli with an S83L mutation in GyrA (GyrA:S83L) emerged in one study from selection at the drug concentration of only 1/230 of the MIC (9). Further stepwise acquisition of additional mutations in the presence of greater drug challenges (10, 11) confers increased resistance to fluoroquinolones (12, 13). The resulting highly resistant forms typically contain a combination of mutations in target genes (GyrAB and/or ParCE), species-specific efflux pumps (such as AcrABC in E. coli), and porins mediating drug influx (14–18).

The observed similarity of intrinsic CIP resistance mechanisms in divergent target pathogens is consistent with the universal mechanism of action of this broad-spectrum drug. Nevertheless, distinct species display different resistibility potentials with respect to the dynamics and extent of acquired resistance. The primary objectives of this study were to assess and compare the dynamics of CIP resistance acquisition and resistance mechanisms in three divergent species representing difficult-to-treat Gram-negative bacteria, Escherichia coli, Acinetobacter baumannii, and Pseudomonas aeruginosa, in a standardized setting of a continuous-culturing device. Among these groups of pathogens, A. baumannii is of particular concern due to its genomic plasticity, a feature which gives rise to diverse isolates displaying preexisting and readily acquired multiple-drug resistance (19, 20). P. aeruginosa, another common nosocomial pathogen, is also known to cause dangerous antibiotic-resistant infections. In our comparative study, we have included the best-studied model Gram-negative bacterium, E. coli K-12, a close relative of clinically relevant pathogenic strains of E. coli.

Although some mutations conferring CIP resistance observed in clinical isolates and laboratory studies of all three target species were previously reported (10, 21, 22), a direct comparison of their evolutionary trajectories is complicated due to different selection conditions. In traditional experimental evolution studies, selection proceeds through a series of bottlenecks reflecting specifics of the experimental setup and bacterial population size. Thus, a serial transfer of small-size bacterial cultures leads to the predominant propagation of high-frequency/low-fitness mutants, while studies of large bacterial populations tend to yield low-frequency/high-fitness mutants that are commonly isolated from patients with CIP-resistant infections (10, 23). Another variable parameter defining the outcome of experimental evolution is the mutant selection window (MSW) (24, 25). The MSW represents a drug concentration range enabling effective elimination of less resistant cells and propagation of more resistant cells. A morbidostat (constant-morbidity) approach used in this study is based on an automated dynamic adjustment of the MSW, shifting upward over the course of experimental evolution (26, 27). This technique allows us to at least partially alleviate selection bottlenecks and enrich evolving bacterial populations with more-robust resistant variants.

**FIG 1:**
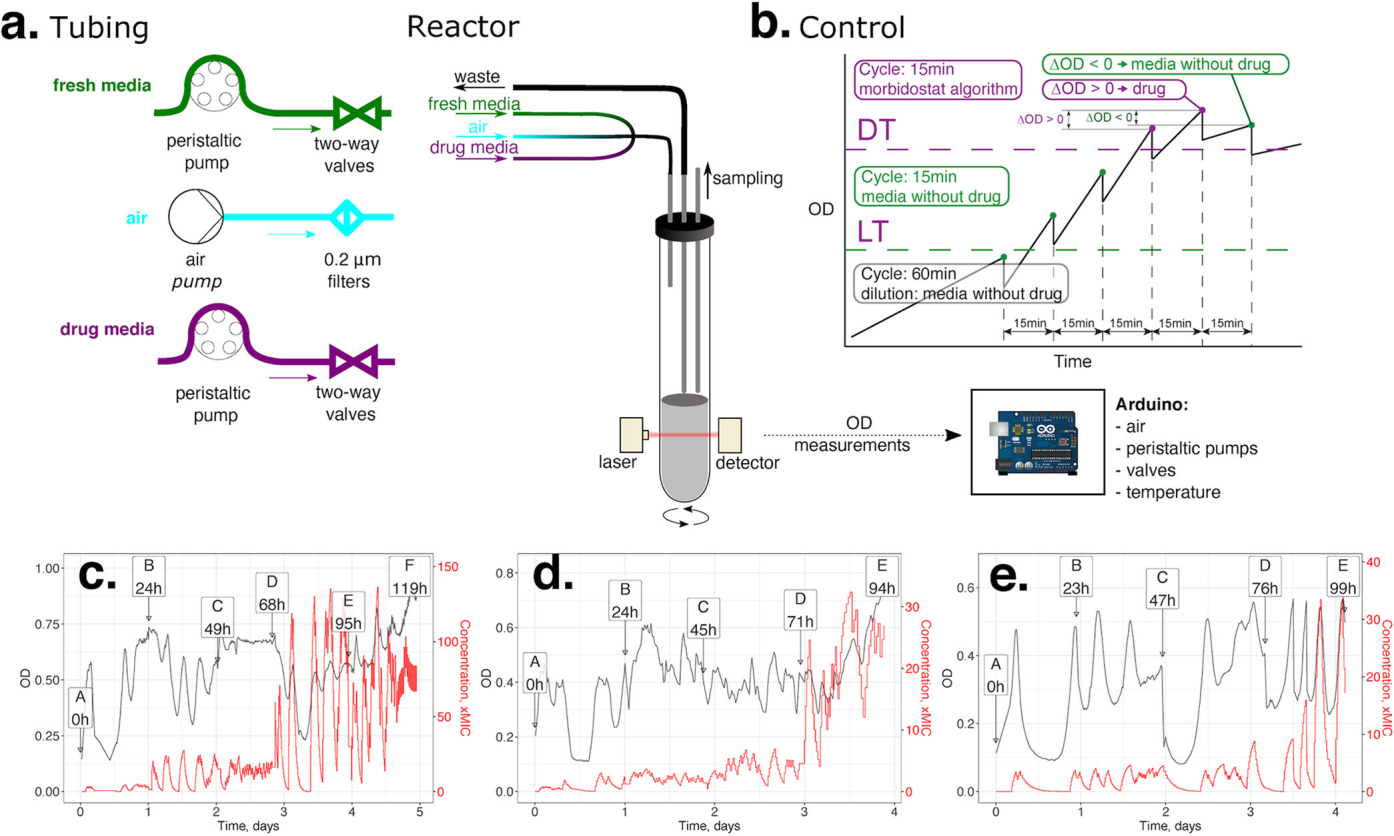
(a to e) Morbidostat design (a), control logics (b), and examples of evolutionary runs of E. coli (c), A. baumannii (d), and P. aeruginosa (e) with ciprofloxacin. (a) Bacterial populations are continuously cultured in a 20-ml glass tube (bioreactor) with magnetic stirring and three input lines: filtered air (blue) and media from two feed bottles, with and without a concentrated drug (purple and green, respectively). The growth (turbidity) is monitored using a laser beam and diode light sensor. Upon periodic addition of 2 to 4 ml medium from the first or the second feed bottle (as defined by control logic, see B), the excess volume is displaced by airflow into a waste bottle. Samples (up to 10 ml) are taken periodically (1 to 2 times per day) through a dedicated sampling port. Our current morbidostat implementation includes 6 parallel bioreactors with individual feed lines that are independently monitored and controlled by the Arduino board with a Windows PC-based user interface. (b) Morbidostat logic is controlled by an Arduino board based on the principles described by Toprak et al. (27) using the real-time OD input from each bioreactor and predefined run parameters: a lower threshold (LT), a drug threshold (DT), and a cycle time (time between dilutions, typically 10 to 20 min when the OD is greater than the LT). Depending on the conditions shown in the diagram, one of the two peristaltic pumps (feeding media with or without drug) are engaged at the beginning of each dilution outgrowth cycle. (c to e) Representative OD profiles (black line) and calculated drug concentration profiles (red line) observed during the course of the experimental evolution of E. coli, A. baumannii, and P. aeruginosa toward resistance against ciprofloxacin (CIP). One of the reactors is shown for each organism, while evolutionary profiles for all other experiments and reactors are provided in Fig. S2. The right axis shows the CIP concentration (times the MIC) as a fold change from the MICs for respective unevolved strains.

For a comparative CIP resistomics analysis of the three selected Gram-negative bacteria in a standardized morbidostat-driven setup (Fig. 1a and b), we leveraged an experimental evolution workflow developed and validated in our previous model study on the evolution of triclosan resistance in E. coli (28). Briefly, the workflow used in this study (see Fig. S1 in the supplemental material) includes (i) competitive outgrowth of six parallel bacterial cultures in a custom-engineered continuous-culturing device, the morbidostat, under gradually increasing antibiotic concentration; (ii) sequencing of total genomic DNA from bacterial population samples taken over a time series; (iii) identification and quantitation of sequence variants (mutations, small indels, mobile elements, insertions) reflecting evolutionary dynamics and inferred resistance mechanisms; and (iv) experimental characterization of genotype-to-phenotype associations via mapping of mutations and determination of MIC values for selected individual clones.

10.1128/mBio.00987-21.2 FIG S1 Morbidostat-based experimental evolution workflow. The initial unevolved culture (step 1) is plated on the agar petri dish (step 2) to make individual colonies. The colonies are collected (step 3) and used to make 6 inoculates (step 4); one colony is used in each reactor for morbidostat cultivation (step 5). From each reactor, samples are taken roughly once in 24 h (step 6). To observe population dynamics (step 8), we perform whole-genome sequencing (WGS) with high coverage for each sample (step 7). The population dynamics is used to choose the optimal number of colonies from plated evolved samples (step 9). For each colony, we do WGS (steps 10 and 11) and MIC tests (step 12) that reveal genotype/phenotype association. Download FIG S1, PDF file, 0.6 MB. Copyright © 2021 Zlamal et al. 2021 Zlamal et al. https://creativecommons.org/licenses/by/4.0/ This content is distributed under the terms of the Creative Commons Attribution 4.0 International license.

10.1128/mBio.00987-21.3 FIG S2 OD profiles (black line) and calculated drug concentration profiles (red line) obtained during the course of the experimental evolution of ciprofloxacin resistance in Escherichia coli BW25113, CEC-2 run (A); Escherichia coli BW25113, CEC-4 run (B); Acinetobacter baumannii ATCC 17978, CAB-1 run (C); Pseudomonas aeruginosa ATCC 27853, PAC-1 run (D); and Pseudomonas aeruginosa ATCC 27853, PAC-2 run (E). The right axis shows the CIP concentration (times the MIC) as a fold change from the MIC values for respective unevolved strains. Sequenced samples are marked with arrows. Download FIG S2, PDF file, 0.6 MB. Copyright © 2021 Zlamal et al. 2021 Zlamal et al. https://creativecommons.org/licenses/by/4.0/ This content is distributed under the terms of the Creative Commons Attribution 4.0 International license.

The combined results from the three analyzed species yielded the identification of nearly all mechanistically and clinically relevant CIP resistance-conferring variants, at least at the level of implicated genes and pathways. This investigation revealed both shared and species-specific aspects of the evolutionary dynamics of resistance acquisition. Despite some differences between morbidostat-deduced mutation profiles and those observed for clinical isolates of individual species, a cross-species comparative resistomics approach allowed us to recapitulate all types of CIP resistance mechanisms. This observation supports the utility of the established workflow for comparative resistomics of known antibiotics, and potentially of novel drug candidates, over a broad range of bacterial pathogens.

## RESULTS AND DISCUSSION

### Evolution of CIP resistance in E. coli BW25113.

Two evolutionary runs were performed to assess the impact of different ranges and rates of CIP concentration escalation on the dynamics and spectra of acquired mutations in E. coli (see Fig. S2A and B in the supplemental material). For both runs, a complete list of significant sequence variants (passing all filters implemented in the computational pipeline) observed in each reactor and time point is provided in Data Sets S2B and C.

Several preexisting low-frequency variants (mostly ∼2 to 3%) detected in the unevolved population at time point 0 were distinct between independently prepared inoculates used in each of the two runs, pointing to their stochastic nature. Most of these variants disappeared from populations over the course of selective outgrowth except two single-nucleotide polymorphisms (SNPs) in genes encoding (i) uncharacterized protein YigI:A146T (BW25113_3820) and (ii) an uncharacterized transporter of the BCCT family, YeaV:V428D (BW25113_1801). These two variants expanded from 3% to 83% of the population in reactor 2 and from 13% to 54% in reactor 6, respectively, by the end of the CEC-2 run; notably, each mutation was apparently coupled with the common GyrA:D87Y mutation (Data Set S2A).

10.1128/mBio.00987-21.7 DATA SET S2 Observed sequence variants (mutations, small indels, and IS element insertions) in the population samples collected and analyzed from five evolutionary runs. (A to E) Sequence variants (nonsynonymous) and their dynamics in evolving populations of E. coli BW25113 (run CEC-2) (A), E. coli BW25113 (run CEC-4) (B), A. baumannii 17978 (run CAB-1) (C), P. aeruginosa (run PAC-1) (D), and P. aeruginosa (run PAC-2) (E). Download Data Set S2, XLSX file, 0.2 MB. Copyright © 2021 Zlamal et al. 2021 Zlamal et al. https://creativecommons.org/licenses/by/4.0/ This content is distributed under the terms of the Creative Commons Attribution 4.0 International license.

**FIG 2:**
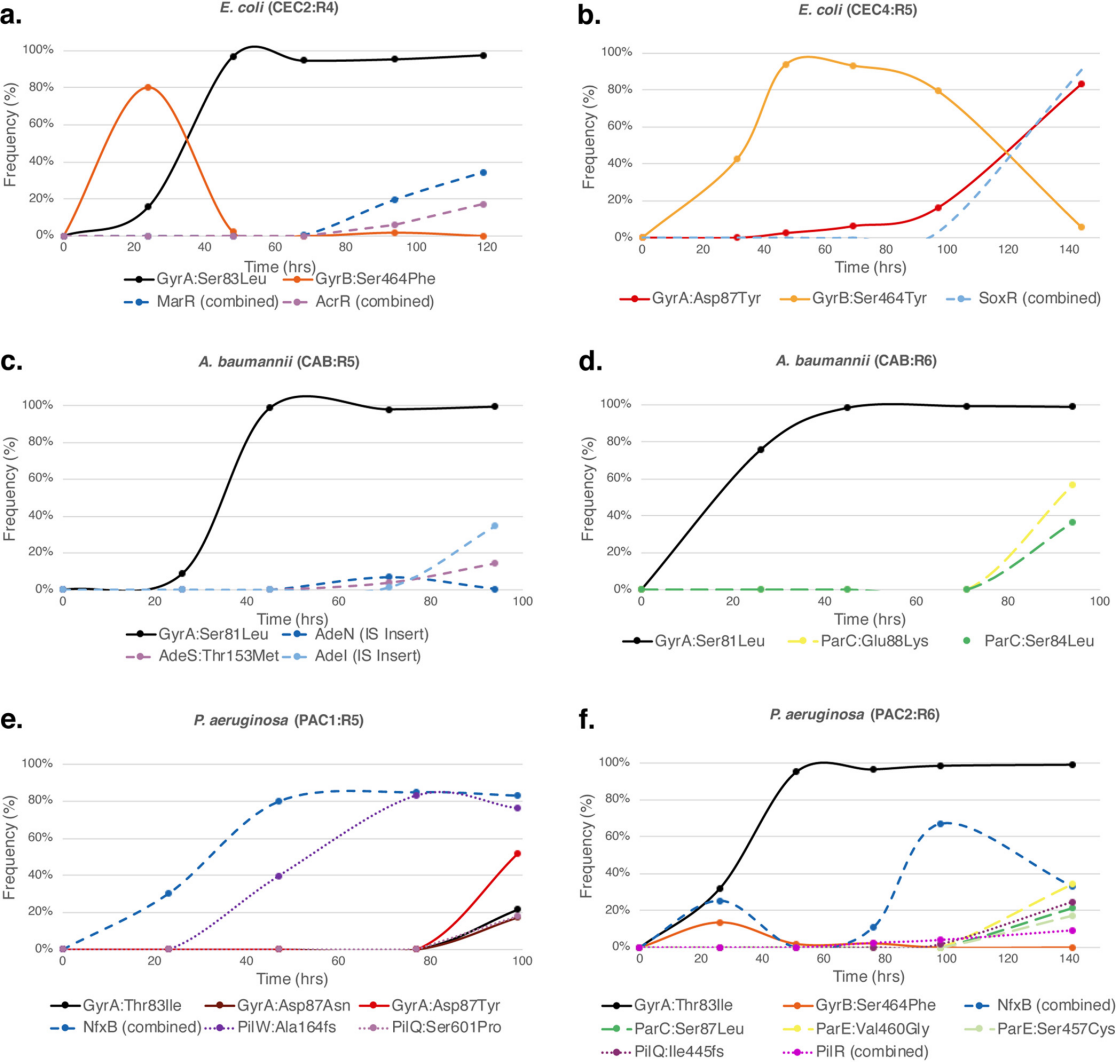
(a to f) Population dynamics of the experimental evolution of ciprofloxacin resistance in E. coli (a, b), A. baumannii (c, d), and P. aeruginosa (e, f). The frequency of major mutations (reaching ≥5%) in evolving bacterial populations is shown as a function of time for selected reactors. The results of selected reactors are shown, as follows: reactor 4 from CEC-2 (a); reactor 5 from CEC-4 (b); reactor 5 from CAB (c); reactor 6 from CAB (d); reactor 5 from PAC-1 (e); and reactor 6 from PAC-2 (f).

The ranges and dynamics of major acquired mutations were generally similar in the two runs, CEC-2 and CEC-4 (Data Sets S2A and B). Of those, the earliest and the most prominent were missense mutations in the A and B subunits of DNA gyrase (GyrA and GyrB), a primary CIP target. These mutations typically emerged within the first 24 h and sustained or further expanded in all populations unless they were outcompeted by other primary target mutations. A variant with the GyrB mutation Ser464Phe, which emerged at an early stage in all six reactors of the CEC-2 run, was rapidly outcompeted by GyrA mutant variants in three of these reactors (Fig. 2a; Fig. S4A). An alternative GyrB mutant variant, the Ser464Tyr variant, dominated all four reactors at an early stage of the CEC-4 run. This variant sustained in all but one reactor (Fig. S4B), where it was outcompeted by a GyrA:Asp87Tyr variant combined with a disruptive deletion in SoxR (Fig. 2b). These distinctive evolutionary trajectories may be driven by a number of factors, including different drug escalation profiles (Fig. S2A and B) and different effects of the two alternative substitutions at GyrB:Ser464 on CIP resistance and fitness.

10.1128/mBio.00987-21.5 FIG S4 Population dynamics of the experimental evolution of ciprofloxacin resistance in Escherichia coli BW25113, CEC-2 run (A); Escherichia coli BW25113, CEC-4 run (B); Acinetobacter baumannii ATCC 17978, CAB-1 run (C); Pseudomonas aeruginosa ATCC 27853, PAC-1 run (D); and Pseudomonas aeruginosa ATCC 27853, PAC-2 run (E). For each reactor in every morbidostat run, the frequency of major mutations (reaching ≥5%) in evolving bacterial populations is shown as a function of time. Download FIG S4, PDF file, 0.6 MB. Copyright © 2021 Zlamal et al. 2021 Zlamal et al. https://creativecommons.org/licenses/by/4.0/ This content is distributed under the terms of the Creative Commons Attribution 4.0 International license.

**FIG 3:**
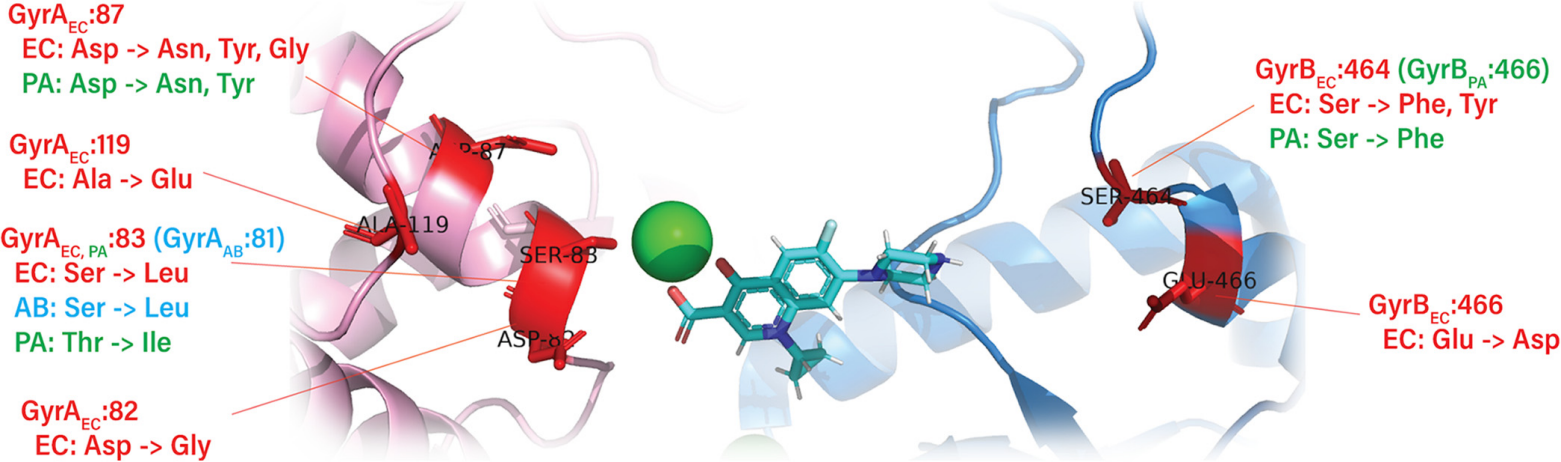
Amino acid substitutions in GyrA (pink chain)/GyrB (blue chain) observed during the course of the morbidostat-based experimental evolution of CIP resistance in E. coli (EC; red text), A. baumannii (AB; blue text), and P. aeruginosa (PA; green text) mapped on a three-dimensional structure (PDB accession number 6RKW). The ciprofloxacin molecule (blue) and Mg^2+^ ion (green) were added by structural alignment of 6RKW with the structure of Mycobacterium tuberculosis gyrase bound to CIP (PDB accession number 5BTC). The substitution equivalent to P. aeruginosa GyrB:Leu128Pro is not shown. It is located near the ATP-binding site of GyrB. Chain A of 6RKW was aligned to chain A of 5BTC using FATCAT. The same rotation translation was then applied to all chains in 6RKW to align the full structure.

The GyrA:Asp87Tyr mutation was among the most prominent variations in GyrA and the only common one between the two E. coli evolutionary runs. Other substitutions at this position included one major variation, Asp87Gly (observed at various frequencies in all but one reactor of the CEC-2 run) and one minor variation, Asp87Asn (observed at low frequency in one reactor of the CEC-4 run). The only other high-frequency GyrA:Ser83Leu variation was observed in one reactor (R4) of the CEC-2 run. These two residues are known to be the most commonly mutated *gyrA* residues in CIP-resistant E. coli (10, 29). Of the two other low-frequency GyrA mutations, the Ala119Glu substitution was previously reported in quinolone-resistant E. coli and Salmonella (10, 30). The second variation, GyrA:Asp82Gly, was also previously observed in these species as well as in CIP-resistant Bartonella bacilliformis (30, 31). All observed amino acid substitutions were located in the vicinity of the known CIP binding site, close to a DNA binding pocket at the interface of the DNA gyrase subunits A and B (Fig. 3). No mutations in the topoisomerase IV subunit A gene *parC*, a known secondary target of CIP, were observed in our study of E. coli, and only one low-frequency mutation, Arg303Ser, was observed in the product of the *parE* gene, encoding subunit B of this enzyme.

Clones representing major GyrA:Ser83Leu and GyrB:Ser464Phe/Tyr mutant variants without any additional mutations were isolated from respective reactors and exhibited 8- to 16-fold increases in CIP MIC values compared to that for the parental (unevolved) strain (Data Set S3A). This magnitude of the effect on CIP resistance is consistent with previous reports (29, 32). Nearly all isolated clones with the GyrA:Asp87Gly variation contained additional mutations leading to efflux upregulation (as described below). In our study, GyrB mutations appear to have a somewhat smaller impact on resistance than GyrA mutations (on average, ∼2-fold), as seen previously (33).

10.1128/mBio.00987-21.8 DATA SET S3 Observed acquired sequence variants and MIC values for selected clones. (A to C) Sequence variants and MIC values for selected clones from the two evolutionary runs of E. coli BW25113 (CEC-2 and CEC-4) (A), from the evolutionary run of A. baumannii ATCC 17978 (CAB) (B), and from the evolutionary run of P. aeruginosa ATCC 27853 (PAC-1, PAC-2) (C). Download Data Set S3, XLSX file, 0.10 MB. Copyright © 2021 Zlamal et al. 2021 Zlamal et al. https://creativecommons.org/licenses/by/4.0/ This content is distributed under the terms of the Creative Commons Attribution 4.0 International license.

**FIG 4:**
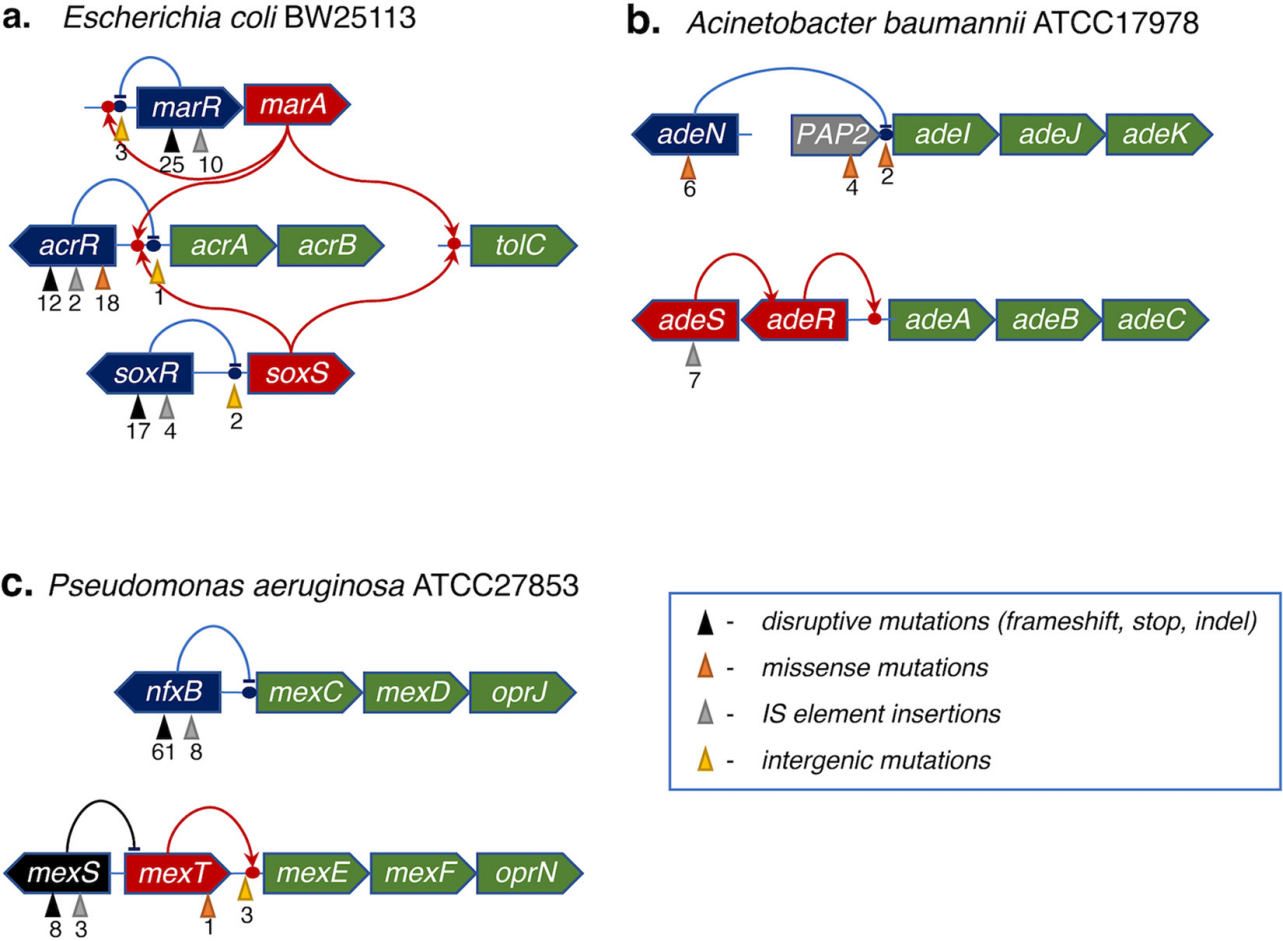
Mutations leading to upregulation of the efflux machinery in Escherichia coli (a), Acinetobacter baumannii (b), and Pseudomonas aeruginosa (c) detected over the course of the experimental evolution of CIP resistance. The total number of distinct variants detected in at least one of the reactors is shown under the color-coded upward-pointing triangles indicating a type of mutation.

Numerous additional mutations emerged in the background of GyrA or GyrB mutant variants at a later stage of experimental evolution, dominated by frameshifts (fs), disruptive deletions, and insertion sequence (IS) elements. These mutations appeared along with an increase of drug concentration, and most of these events are predicted to lead to upregulation of the efflux machinery. The most prominent in both runs were the loss-of-function events affecting the transcriptional regulators MarR, AcrR, and SoxR, which negatively control the expression of the well-known efflux pumps MarAB and AcrAB and an outer membrane protein, TolC (Fig. 4a). Disruptive mutations occurred in over 30 distinct locations in the gene *marR* (Data Sets S2A and B). Similar events were shown previously to increase MarA expression, thus contributing to CIP resistance and to a broader multidrug resistance phenotype (11, 34). Clinical E. coli isolates with fluoroquinolone resistance commonly contain mutations in *marR*, causing constitutive expression of the *mar* operon (35). Deletion of the C terminus of MarR increased E. coli CIP resistance *in vivo* (36), and inactivation of MarR has been shown to increase *marA* expression, effecting drug efflux by way of the transcriptional amplification of *acrAB* and *tolC* pumps (17, 37).

Among several missense mutations (observed only in the CEC-2 run), the most prominent amino acid substitution, MarR:Thr39Ile (which expanded to up to 1/3 of the bacterial population when combined with GyrA:Asp87Tyr in reactor R2), affected the known DNA-binding site of the MarR repressor (38). Dynamics of acquisition of MarR mutational variants appear to have been faster in the CEC-2 run than in the CEC-4 run (after 48 h versus 96 h, respectively), reflecting the sharper increase in drug concentration in the CEC-2 run. The MICs for clones with *marR* mutations combined with *gyrA* or *gyrB* mutations were 4- to 8-fold higher than those for corresponding clones with mutations only in DNA gyrase (Data Set S3A).

IS element insertions, frameshifts, and deletions occurred in multiple positions of both the *acrR* gene, encoding a transcriptional regulator of the *acrAB* operon, as well as in the intergenic region of the *acrA<>acrR* divergon (Fig. 4a). These variants appeared at relatively low frequencies at the latest stage of experimental evolution, after ∼90 h in both runs (Fig. 2a; Fig. S4A and B). Among isolated clones, *acrR* mutations were found only combined with mutations in *marR* and one of the DNA gyrase subunits (*gyrA* or *gyrB*). The MIC values for these triple mutants were the highest observed in this study; they were up to 128-fold higher than the MIC for the wild-type strain (Data Set S3A).

Point mutations, frameshifts, and deletions also occurred frequently in *soxR*, the redox-sensitive repressor which negatively regulates SoxS, the transcriptional activator of efflux genes *acrAB* and *tolC* (Fig. 4b). The SoxR mutation Gly121Asp, which occurred in the CEC-2/R5 population, has recently been shown to increase SoxS expression 12-fold (34). As with *marR*, mutations in *soxR* emerged between 48 and 96 h, more rapidly in CEC-2 than CEC-4 (Fig. 4b; Fig. S4A and B). Deletions and small insertions in *soxR* were found in clones along with DNA gyrase mutations, contributing to a further increase in the MIC by a factor of 4 to 8 (Data Set S3A).

Despite the relatively low abundance of individual mutations in the efflux regulators *marR*, *acrR*, and *soxR*, a very high combined frequency of efflux-upregulating mutations in both CEC-2 and CEC-4 makes increased efflux a predominant mechanism for elevating CIP resistance in the background of *gyrAB* mutations. This trend is consistent with other published studies, including laboratory evolution experiments and analysis of CIP-resistant clinical isolates of E. coli (23, 39). A single major deficiency observed in the study with E. coli (but not with two other species [see below]) is the failure to recover ParC variations that are typically found as GyrA and ParC double mutations in clinical isolates. This may reflect a lower intrinsic mutation frequency of our model E. coli K-12 strain than those of pathogenic strains of E. coli.

### Evolution of CIP resistance in Acinetobacter baumannii ATCC 17978.

To accurately assess preexisting variations that occur at much higher frequency in A. baumannii than in E. coli K-12, we first sequenced and assembled a complete genome corresponding to our stock of A. baumannii ATCC 17978 (ENA project number PRJEB36129). This assembly was further used as a reference for this study and featured substantial differences from publicly available sequences: 16 variations from the GCA_001593425 assembly and 87 variations from the GCA_000015425.1 assembly (at a >85% frequency threshold) over 98.5% and 99.0% mapped reads, respectively. To minimize a clonal bias, which could have resulted from preexisting variations, we prepared each of the starter cultures from individual colonies and sequenced total genomic DNA isolated from these cultures (samples A1 to A6). To account for potential genomic rearrangements, we used a hybrid approach combining the data from high-coverage Illumina sequencing (short reads) with Oxford Nanopore sequencing (long reads). This approach revealed that, in contrast to the public genomes, all starting cultures have pAB3 (40) as an extrachromosomal plasmid (in public genomes, it is integrated into the chromosome) and an additional 52-kb locus (Data Set S1C).

10.1128/mBio.00987-21.6 DATA SET S1 Read alignments in the genomes of E. coli, A. baumannii, and P. aeruginosa. (A) Read alignment statistics for population samples; (B) read alignment statistics for clonal samples; (C) genes comprising a mapped large deletion in the A. baumannii ATCC 17978 chromosome. Download Data Set S1, XLSX file, 0.07 MB. Copyright © 2021 Zlamal et al. 2021 Zlamal et al. https://creativecommons.org/licenses/by/4.0/ This content is distributed under the terms of the Creative Commons Attribution 4.0 International license.

The single A. baumannii evolutionary run was performed using a mild drug escalation regimen which started from a CIP concentration in pump 2 corresponding to 1.25 times the MIC. The dynamics of CIP resistance acquisition observed in five parallel reactors appeared faster in the case of A. baumannii than in the case of E. coli (Fig. 1d; Fig. S2C). One reactor (R1) was excluded due to technical failure. Resistance was driven at the first stage by a single *gyrA* mutation resulting in Ser81Leu, which was first detected after day 1 and expanded to ≥95% by day 3 (Fig. 2c and d; Data Set S2D). This mutation leads to an ∼8-fold increase in the MIC (Data Set S2B), which is similar to the impact of the position-equivalent GyrA:Ser83Leu variant of E. coli (Data Set S2A). The same mutation is commonly found in CIP-resistant A. baumannii isolates and has a similar impact on the MIC, as reported for A. baumannii ATCC 19606 (41).

At the next stage of experimental evolution, adaptation to higher drug levels was attained via a variety of mutations apparently upregulating the drug efflux machinery (Fig. 2c and 4b; Fig. S4C; Data Set S2C). Such upregulation was achieved with three classes of variants: those that exhibited (i) inactivation of the transcriptional repressor AdeN of the efflux pump AdeIJK, caused exclusively by the insertion of different mobile elements (IS*Ab1*, IS*Ab11*, and IS*Ab12*); (ii) polar effects from the insertion of IS elements (IS*Ab1*, IS*Ab12*, IS*Ab18*, and IS*Abcsp3*) in the intergenic region or in the gene encoding membrane-associated phospholipid phosphatase immediately upstream of the *adeI* gene; and (iii) several missense mutations in the AdeS sensory histidine kinase of a two-component system (AdeSR) positively regulating another efflux pump, AdeABC (42–44). IS element insertions upstream of the *adeIJK* operon were previously reported to reduce the susceptibility of A. baumannii to ciprofloxacin and other substrates of the respective efflux pump (45, 46). Notably, one of the AdeS mutant variants, AdeS:Asp60Gly, expanded up to 65% in reactor R3’s population by the end of day 4. An apparent prevalence by abundance of AdeABC- versus AdeIJK-upregulating variants (Table 1) is consistent with a recent report on the higher efficiency of the AdeABC transporter than of the AdeIJK transporter in the efflux of hydrophilic agents, such as fluoroquinolones (47).

**TABLE 1:** Major mutational variants detected in the course of the experimental evolution of ciprofloxacin resistance^*a*^

Target(s)	E. coli			A. baumannii			P. aeruginosa		
	Variant	Max. freq. (%)^*b*^	No. of reactors (out of 10)	Variant	Max. freq. (%)^*b*^	No. of reactors (out of 5)	Variant	Max. freq. (%)^*b*^	No. of reactors (out of 12)
Primary									
GyrA, DNA gyrase subunit A	Ser83Leu	98	1	Ser81Leu	99	5	Thr83Ile	100	12
	Asp87Asn	17	1				Asp87Asn	79	2
	Asp87Tyr	99	4				Asp87Tyr	52	1
	Asp87Gly	74	6						
	Ala119Glu	7	2						

GyrB, DNA gyrase subunit B	Ser464Phe	96	6				Leu128Pro	7	1
	Ser464Tyr	99	4				Ser466Phe	63	4


Secondary									
ParC, topoisomerase IV subunit A				Ser84Leu	36	4	Ser87Leu	53	6
				Glu88Lys	86	2			
				Asn334Tyr	4	1			

ParE, topoisomerase IV subunit B							Leu121Pro	8	1
							Ser457Gly	6	1
							Ser457Cys	17	1
							Ser457_insArg	9	1
							Val460Gly	62	2

Efflux pump deregulation	*marR*^*c*^	86	9	*adeN*^*c*^	28	5	*nfxB*^*c*^	90	12
	*soxR*^*c*^	95	9	*adeS*^*c*^	72	4	*us_mexE*	39	1
	*us_soxS*	10	2	*us_adeI*	36	4	*us_mexT*	12	5
	*acrR*^*c*^	63	9				*mexT*:G58D	6	1
	*us_acrA*	53	3				*mexS*^*c*^	94	7

Other strongly implicated genes							*pilBOPQRSTWZ*^*c*^	98	11
							*mutL*^*c*^	37	2
							*mutS*^*c*^	27	3

a Listed mutations that reached ≥5% in at least in one reactor and one time point.

b Maximum observed frequency reached at any time point in any reactor.

c Disruptive mutations (mainly frameshifts, stops, small indels, and IS elements).

The upregulation of the efflux pump AdeIJK in the background of the GyrA:Ser81Leu variant led to an additional 2- to 4-fold increase in the MIC (Data Set S3B). This was determined for individual clones representing mutant variants of both types: those with IS insertions in the *adeN* gene and those with IS insertions in the intergenic region upstream of gene *adeI* (16 and 32 times the MIC, respectively, for the unevolved strain). No mutations were observed in genes associated with yet another known multidrug efflux system of A. baumannii, AdeFGH (48).

Finally, the highest level of CIP resistance was achieved as a result of a combination of the initial GyrA:Ser81Leu variant with additional missense mutations in the *parC* gene, encoding DNA topoisomerase IV subunit A, a secondary target of fluoroquinolone drugs. These mutations emerged only at the latest stage of experimental evolution (day 4) (Fig. 2d; Fig. S4C). The ParC:Asn334Tyr variant reached low frequency (4% in R2) only in one reactor, whereas two other variants (with ParC:Ser84Leu and ParC:Glu88Lys mutations) emerged in several reactors, reaching much higher frequencies (up to 86%) (Data Set S2C). Both variants exhibited resistance 128-fold that of the unevolved strain, the highest increase in MIC observed with individual clones (Data Set S3B). These two mutated residues are position-equivalent to the most commonly mutated Ser80 and Glu84 residues in E. coli ParC (41, 49, 50). A commonly reported CIP-resistant mutation, Ser458Ala, in the topoisomerase IV subunit B gene *parE* (51) was observed at the last time point in only one reactor (R1) at low abundance (4%). Notably, MIC values for all characterized double mutant variants (2.5 to 20 mg/liter) are substantially higher than a clinical breakpoint (1 mg/liter) reported for Acinetobacter by EUCAST (https://www.eucast.org/clinical_breakpoints/), as marked in Data Set S3B.

### Evolution of CIP resistance in Pseudomonas aeruginosa ATCC 27853.

To further expand the comparative resistomics approach and assess common and species-specific trends in the dynamics of acquisition and mechanisms of CIP resistance, we applied the optimized morbidostat-based experimental evolution workflow to study another important Gram-negative pathogen, Pseudomonas aeruginosa. Two evolutionary runs (PAC-1 and PAC-2) with P. aeruginosa ATCC 27853 were performed using starter cultures prepared from six distinct colonies. Unlike with A. baumannii, this analysis did not detect any substantial variations between these cultures (Data Sets S2D and E). Some of the starter cultures contained up to 20 preexisting low-frequency variants (in a range of 1 to 10%). These low-frequency variants reflect stochastic microheterogeneity, and they typically disappeared from bacterial populations after day 1 of selective outgrowth in the morbidostat (Data Sets S2D and E).

A technical challenge originated from a tendency of P. aeruginosa to form a visible biofilm on the glass surface of the reactor, especially located near the interface with air. This problem was mitigated by keeping culture densities well within logarithmic growth phase (optical density at 600 nm [OD_600_] of ≤0.55) and by daily transfer of cultures to clean reactors, which was performed along with sample collection.

Using an improved version of morbidostat software allowed us to perform two evolutionary runs with more flexible iterative modulation of drug concentration during the runs. The second run (PAC-2) employed a shallower drug escalation mode in the early stage (days 1 to 3) and enhanced the overall duration of the experiment (6 days instead of 4 days for PAC-1) with a larger number of collected and analyzed samples (Fig. S4D and E). In contrast to the case of E. coli, various critical parameters of morbidostat runs with P. aeruginosa appeared to have some notable impacts on the range and dynamics of acquisition of certain mutational variants, as outlined below (Fig. 2e and f; Fig. S4D and E).

The most prominent primary mutation appearing in all reactors of both the PAC-1 and PAC-2 runs was the GyrA:Thr83Ile variant (Table 1), which is equivalent to GyrA:Ser83Leu in E. coli and Ser81Leu in A. baumannii. This was the only GyrA variant in the PAC-2 run, and it emerged typically on day 2 (in four out of six reactors) and expanded to up to ∼100% of the population by days 3 to 4 in all reactors. The same GyrA:Thr83Ile variation was also universally present and dominant in all six reactors of the PAC-1 run. However, it appeared at a somewhat later stage (typically on day 3), in most cases after disruptive mutations in the *nfxB* gene (see below). Additional variants, with GyrA:Asp87Asn and GyrA:Asp87Tyr, appeared in two reactors of the PAC-1 run and partially outcompeted the GyrA:Thr83Ile variant by the end of the run (Fig. 2e and f; Fig. S4D and E). Two of these three variants (GyrA:Thr83Ile and GyrA:Asp87Asn) represent the two most commonly mutated positions in CIP-resistant P. aeruginosa reported for both clinical and laboratory isolates (52, 53).

The only prominent GyrB:Ser466Phe variation (the equivalent of the GyrB:Ser464Phe variation observed in E. coli) was detected transiently in one reactor in each run (R6 of PAC-1 and R5 of PAC-2), peaking at 30% and 63% of respective populations, only to be entirely outcompeted by GyrA:Thr83Ile-containing variants by the end of both runs.

Among the most striking differences observed between the two runs was the appearance of mutations in the genes *parC* and *parE*, encoding subunits A and B of DNA topoisomerase IV exclusively in PAC-2. Remarkably, the most prominent mutation, ParC:Ser87Leu, appeared in all six reactors on the last day of the run, when the drug concentration was highest, reaching up to 53% of the population as a secondary mutation in the background of GyrA:Thr83Ile-containing variants (Fig. 2f; Fig. S4E). Among more diverse (albeit less universal) ParE mutations, the most prominent were ParE:Val460Gly and ParE:Ser457Cys, both of which reached their highest abundances (34% and 17%, respectively) in the same reactor (R6) on the last day of the PAC-2 run (Fig. 2f). These exhibited the same general evolutionary dynamics as ParC variants and also emerged in the background of the GyrA:Thr83Ile mutation (Fig. 2f).

Among the mutational variations driving efflux upregulation, the most common and abundant were various types of disruptive mutations in the *nfxB* gene, encoding a transcriptional repressor of the *mexCD-oprJ* operon (54, 55) (Fig. 4e and f). Disruptive mutations in the *nfxB* gene were reported to have pleiotropic effects improving fitness and antibiotic resistance, contributing to the lowered expression of outer membrane porins and improved drug efflux (16, 56). In our study, the entire range of 61 mutations unambiguously leading to a loss of function (frameshifts, nonsense mutations, and indels) and 8 missense mutations were found spread over all 12 reactors in both PAC runs, collectively reaching from 35% to 90% abundance at a minimum of one time point in every reactor (Fig. S4D and E). However, the dynamics of their appearance and accumulation were strikingly different between the two runs. Indeed, in nearly all reactors of the PAC-2 run (except R3), these mutations emerged and accumulated after or together with the driving GyrA:Thr83Ile variation, similar to the evolutionary dynamics patterns observed for E. coli and A. baumannii. In contrast, in all reactors of the PAC-1 run, NfxB mutational variants reached >30% overall abundance prior to comparable accumulations of GyrA variants that emerged later and expanded in the background of NfxB and/or other adaptive mutations (Fig. S4D; Data Set S2D). This observation provides another example of how the difference in drug escalation regimen may affect evolutionary trajectories of CIP resistance in P. aeruginosa.

Most of the individual NfxB-disruptive variants in the PAC-2 run were present at relatively low frequencies (2 to 20%) and randomly distributed among reactors. Remarkably, one NfxB variant, with a replacement of the stop codon by a cysteine codon, which resulted in extension of the NfxB protein by 68 amino acids, appeared in all six reactors in the background of the GyrA:Thr83Ile variant, peaking at up to an 85% abundance in the population (Data Set S2E). In every case, the abundance of this variant shrank to <20% on the last day of the run. This mutation was characterized earlier and found to lead to substantial overexpression of the MexCD-OprJ efflux pump (57). A similar, but less contrasted, picture was observed during the course of the PAC-1 run, in which the same variant appeared transiently in the middle of the run in five out of six reactors, reaching 10 to 20% abundance, after which it completely disappeared from populations by the end of the run (Data Set S2D). The single-most-abundant disruptive NfxB:Ile65fs variant reached >80% frequency in the population and remained until the end of the run, coupling with at least two out of three GyrA variants that emerged in this reactor (R5) only on the last day of the PAC-1 run.

Additional low-frequency mutations potentially upregulating another multidrug efflux pump, MexEF-OprN (58), were detected in the upstream region of the respective operon (typically at a later stage [see Data Sets S2D and E]). One of these was a missense mutation (Gly258Asp), and two were intergenic mutations in the upstream region of the gene *mexT*, encoding its transcriptional activator (Fig. 4c). However, the largest variety of mutations reaching up to 85% in all but one reactor in PAC-1 (and one reactor in PAC-2) were found within the sequence of a gene encoding an uncharacterized oxidoreductase, MexS, located in the divergon with MexT, immediately upstream of *mexE*, the first gene of the *mexEF-oprN* operon (Fig. 4c). It was previously shown that a transposon inactivation of the *mexS* gene leads to upregulation of this typically quiescent operon via a yet-unknown mechanism (59). Single amino acid substitutions in MexS leading to MexT-driven activation of the *mexEF-oprN* operon are frequently found in clinically isolated *nfxC* mutants of P. aeruginosa (a general term for strains with an upregulated MexEF-OprN efflux pump) displaying enhanced virulence and drug resistance (60). Both the MexCD-OprJ and MexEF-OprN systems affected in this study are known to be primary drivers of fluoroquinolone resistance; no mutations were found in several other known efflux systems of P. aeruginosa (61).

The last type of frequent mutations (including frameshifts, indels, and IS elements) was observed at the late stage of both the PAC-1 and PAC-2 evolutionary runs in several *pil* genes involved in type IV pilus and fimbria biogenesis/assembly (Table 1; Data Sets S2D and E). Some of these mutations expanded to high abundance when coupled with GyrA mutational variations, e.g., up to 88% for PilQ:Gln232fs (R1 in PAC-1) and 65% for PilS:Gln14fs (R4 in PAC-2). Notably, a mutant with PilW:Ala164fs appeared (in R5 of PAC-1) on day 2 in the background of the NfxB:Leu62fs variant (in the absence of any GyrA/B mutations). The NfxB:Leu62fs PilW:Ala164fs double mutant expanded to ∼85% of the population on day 3 and provided a genetic background for the appearance of the three GyrA variants on day 4 (Fig. 2c; Data Set S2D). The loss of type IV pili has been observed previously under CIP stress in P. aeruginosa (62). While the mechanistic rationale for this class of events remains unclear, it was hypothesized that the loss of type IV pili, known receptors for filamentous phages implicated in chronic infection, contributes to resistance against superinfection and lysis under ciprofloxacin stress (63).

The analysis of representative clones selected from PAC-1 confirmed the existence of CIP-resistant NfxB mutational variants lacking target-based mutations. Such clones exhibited a ≤16-fold increase in the MIC, comparable to the observed impact of mutations in GyrA or GyrB (Data Set S3C). A combination of GyrA mutations with the loss of NfxB and/or MexS increased the CIP resistance of respective isolated clones to 64-fold the MIC for the unevolved strain. Notably, additional mutations in *pil* genes detected in most of the selected clones do not appear to have any additional impact on the MIC for respective variants (e.g., compare the GyrA MexS PilO or GyrA MexS PilQ variant with the GyrA MexS variant in Data Set S3C). Moreover, a single isolated clone featuring a loss of PilT in the absence of any other driver mutations displayed a MIC equivalent to that for the unevolved strain, supporting a conjecture that mutations in *pil* genes are more likely to contribute to fitness rather than to CIP resistance. The strongest increase in the MIC, to 128-fold, was observed for the GyrA ParC and GyrA ParE double mutants. As in the case of A. baumannii, MIC values for nearly all characterized clones were above a clinical breakpoint (0.5 mg/liter) reported for Pseudomonas by EUCAST (https://www.eucast.org/clinical_breakpoints/), as marked in Data Set S3C.

Another distinctive feature observed in the experimental evolution of P. aeruginosa was the emergence of disruptive mutations in the *mutS* and *mutL* genes, encoding DNA mismatch repair proteins. The appearance of MutS frameshift variants in R5 at the very end of PAC-1 run coincided with a spike of mutations in the same abundance range (Data Set S2D). More remarkable is a simultaneous occurrence of both mutational variants, the MutS:Ala358fs (27%) and MutL:Leu706Arg (31%) variants, in the same reactor (R4) on the last day of the PAC-2 run. Despite having similar abundances, these two variants likely represent two distinct clonal subpopulations, each accompanied by a broad range of secondary mutations (Data Set S2E). Most of the accompanying secondary mutations did not occur at any other time point or in any other reactor. The loss of MutS function is known to increase the frequency of DNA replication errors leading to an explosion of mutations, as demonstrated in many bacteria, including P. aeruginosa (64). While limited to a single reactor per run, such a trajectory is not uncommon for P. aeruginosa, which was reported to acquire *mutS* loss-of-function mutations in cystic fibrosis patients possibly accelerating adaptation to the host environment and acquisition of antibiotic resistance (65). That said, the actual impact of *mutS mutL* disruption on the evolution of CIP resistance in our studies is not obvious. As already mentioned, MutS and MutL variants appeared only on the last day of each evolutionary experiment, whereas multiple coappearing mutations seemed unrelated to drug resistance. Based on the abundance, only the GyrA:Asp87Asn variant (17%), which also emerged in R5 at the last time point of the PAC-1 run, could have coappeared in the background of the MutS:Arg302fs variant. The analysis of five isolated clones featuring GyrA ParC MutL variants revealed the presence of from 50 to 80 mostly nonoverlapping additional mutations (tabulated in the lower part of Data Set S3C). Of the total 275 mutations, only six fell into potentially relevant genes associated with efflux pumps (*mexB*, *mexE*, *mexW*, and *mdtC*). Notably, for all these clones, the MIC values were comparable to those for GyrA ParC double mutants over notably impaired fitness (growth rate).

### Comparative resistomics: shared and unique features of evolutionary trajectories to CIP resistance in E. coli, A. baumannii, and P. aeruginosa.

**FIG 5:**
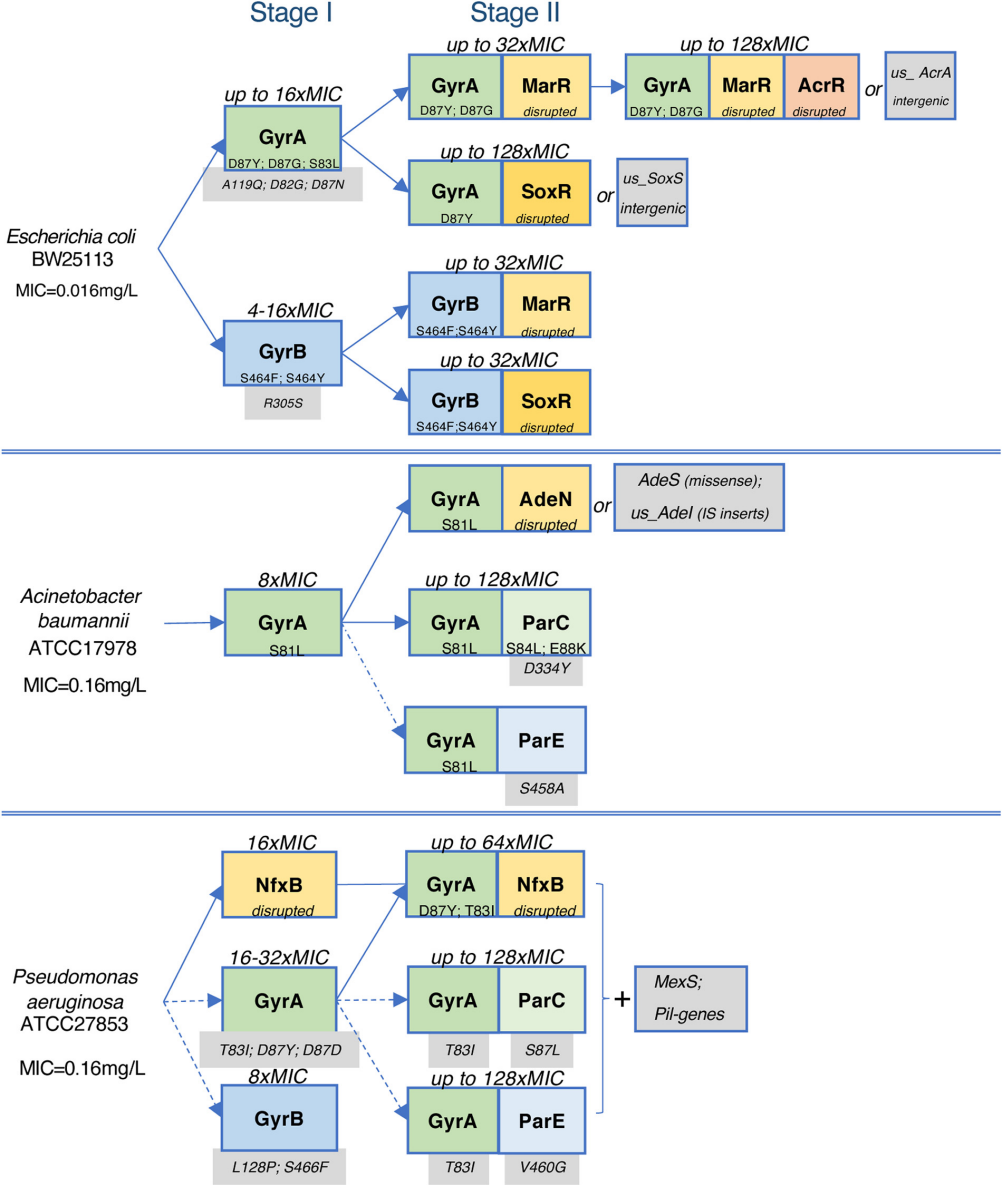
Trajectories and stages in the experimental evolution of CIP resistance in E. coli (top), A. baumannii (middle), and P. aeruginosa (bottom). Major driver mutations are shown in color-coded boxes, with missense mutations in targets (green and blue) and disruptive mutations in efflux regulators (yellow and brown). Additional potentially relevant mutations detected in populations but not in isolated clones are shown on a gray background.

The present experimental evolution studies provided a foundation for comparative resistomics analysis of three representative Gram-negative bacterial species. Observation of each species in standardized continuous-culturing conditions permitted discernment of common and species-specific aspects of evolutionary trajectories toward CIP resistance (Fig. 5). The observed variety of these evolutionary trajectories can be approximated by a largely shared two-stage process. In stage I, when the drug pressure was moderate (typically, days 1 to 3 of the morbidostat run), the emerging resistance (typically, 4- to 16-fold the MIC for the unevolved parental strain) was usually driven by a single mutation rapidly expanding over the entire bacterial population in each reactor (Fig. 1c to e; Fig. S2). In all reactors of E. coli (CEC-2 and CEC-4), A. baumannii (CAB-1), and one of the two morbidostat runs of P. aeruginosa (PAC-2), the earliest (stage I) mutations occurred in one of the two subunits of DNA gyrase (GyrA or GyrB). Among them, the most prominent and sustainable were amino acid substitutions in two positions of GyrA: Ser/Thr83 or Asp87 (by numeration of E. coli GyrA). At least one of these GyrA variants ultimately appeared in nearly all reactors, expanding to 100% abundance by the end of each run (Table 1; Data Sets S2A to E). Not surprisingly, these amino acid residues located in the CIP binding site (Fig. 3) are the positions of the most common mutations in CIP-resistant clinical isolates (10, 29). Of these two positions, the former was the only one affected in A. baumannii (5 out of 5 reactors) and the most universal (in 12 out of 12 reactors) among the two affected positions in P. aeruginosa GyrA. Amino acid substitutions at this position were shown to substantially reduce drug binding to the GyrAB-DNA complex (66) and thus confer a higher increase in MIC than other *gyrA* mutations (33). Notably, the GyrA:Ser83Leu variant appeared in only one out of 10 reactors of E. coli runs (Table 1), while most other reactors were dominated by GyrA:Asp87 (Tyr, Gly, or Asn) variants.

Another distinctive feature of evolution of CIP resistance in E. coli was a somewhat unexpected prominence of GyrB mutational variants. Thus, GyrB:Ser464Phe and GyrB:Ser464Tyr emerged as the earliest stage I variants in all 6 reactors of CEC-2 and all 4 reactors of CEC-4 (Table 1; Fig. S4A and B). Moreover, they sustained their predominant abundance until the end of both runs in 7 out of 10 reactors via coupling with efflux-deregulating mutations in stage II. While GyrB is not considered a direct target of fluoroquinolones, the affected Ser464 position is located close to the CIP binding site in the GyrA-GyrB complex (Fig. 3) (67). Further, CIP resistance-conferring mutations in this position were previously reported for Citrobacter freundii, Morganella morganii, Salmonella enterica serovar Typhimurium, and P. aeruginosa (68–71). Notably, no GyrB variants were observed in A. baumannii, and a position-equivalent GyrB:Ser466Phe variant appeared only transiently in P. aeruginosa morbidostat runs (Table 1; Fig. S4D and E).

Important similarities along with interesting differences can be deduced from the comparative analysis of mutational profiles of these three bacterial species with respect to a known secondary target of fluoroquinolones, DNA topoisomerase IV, comprised of two subunits (ParC and ParE). Unlike with A. baumannii and P. aeruginosa, no mutations were detected in the *parC* or *parE* gene in either one of the two E. coli morbidostat runs (Table 1). Moreover, even in the other two species, such mutations were relatively rare and occurred exclusively in stage II in the background of stage I-born GyrA mutations (Fig. S4C to E). An interesting difference between these species is that while A. baumannii featured only ParC variants, the second P. aeruginosa morbidostat run (PAC-2, but not PAC-1) revealed a substantial representation of both ParC and ParE variants (Table 1). Despite a clear distinction of mutational profiles and frequencies between the primary (GyrA/B) and secondary (ParC/E) CIP targets, the complete lack of ParC/E mutational variants in evolutionary experiments with E. coli is somewhat surprising given the reported presence of such mutations in CIP-resistant clinical isolates of E. coli (51, 72). This discrepancy may reflect certain limitations of the morbidostat-based approach for modeling clinical evolution. On the other hand, the failure to observe these low-probability variants in the laboratory strain of E. coli K-12 used in our study may also have originated from a lower intrinsic mutation frequency than in clinically relevant strains of pathogenic E. coli.

In addition to a narrow set of missense mutations in universal target genes, numerous different but mostly disruptive mutations emerged in a variety of efflux-regulating genes in all three species examined (Table 1; Fig. 4). Among the common features of these mutational events, (i) they typically occurred in stage II in the background of already-accumulated GyrA/B variants (the universal evolutionary trajectory in E. coli and A. baumannii and the predominant one in P. aeruginosa) and (ii) the most common targets of these mutations in all three species were negative regulators (transcriptional repressors) of efflux pump operons. Not surprisingly, a large fraction of such mutations includes nonsense mutations (stops), frameshifts, small indels, and IS elements (Fig. 4) representing a clear loss of function. Notably, the last of these was the most common, if not the only type of genetic alteration leading to the loss of gene function in A. baumannii. In E. coli, IS elements comprised more than half of the loss-of-function variants of the negative efflux regulator AcrR (but not MarR or SoxR) and two intergenic variations potentially leading to derepression of the positive efflux regulator SoxS. Less frequent mutational events with the same type of downstream effects (upregulation of efflux pumps) occurred in intergenic regions (possible binding sites of respective transcriptional regulators). Additionally, several exclusively missense mutations arose in positive regulators (AdeS in A. baumannii and MexT in P. aeruginosa).

A notable deviation from the nearly universal evolutionary trajectory (target first, efflux later) was observed in P. aeruginosa for NfxB, a transcriptional repressor of the *mexCD-oprJ* efflux operon. NfxB-inactivating mutations emerged in both stage I (in the absence of any target mutations; in PAC-1 but not in PAC-2) and stage II of CIP resistance evolution in P. aeruginosa.

All major types of CIP resistance-conferring mutations described in the literature were observed in at least one of the three species in our study. These results support the utility of the established morbidostat-based workflow to elucidate antibiotic resistance mechanisms in a comprehensive manner. Strengthened further by a comparative resistomics approach, this study allowed us to elucidate and generalize major pathways to CIP resistance in a group of divergent Gram-negative bacterial pathogens.

### Concluding remarks.

We employed a comparative dynamic analysis of genetic adaptation to reveal both shared and distinctive features of three divergent Gram-negative bacterial species’ evolutionary trajectories toward CIP resistance. Despite obvious differences between the experimental conditions in the morbidostat and the complexity of processes leading to drug resistance in bacterial infections, the results obtained in this study are generally consistent with those deduced from clinical CIP-resistant isolates.

Many studies have suggested that resistance mutations observed in clinical specimens are biased toward low fitness costs, as fitness may be more important than the extent of resistance under clinical conditions (10, 39, 73). This observation is perhaps best exemplified by the apparent bias against *marR* mutations in ciprofloxacin-resistant clinical isolates. The interplay of fitness and resistance may explain the general mutation dynamics observed in this study. An initial emergence of rare target mutations with a presumably low fitness cost occurred in the morbidostat, similar to what is observed in the clinic. This was followed by an explosion of various efflux regulator mutations later in the evolutionary trajectory, as high drug resistance became the primary hurdle and additional adaptation mechanisms had to be engaged for survival (10). Especially in human infection, early selection appears to favor the mutations causing the lowest fitness detriment, even when the resulting increase in resistance is relatively modest (39).

Indeed, disruptive mutations observed abundantly in efflux regulator genes in our study are statistically much more likely to occur than a few beneficial amino acid substitutions in a very limited set of positions in CIP target enzymes, and yet, such mutations were observed mostly as secondary events at a later stage (stage II) of experimental evolution in the morbidostat. This is consistent with the observations that these mutations in clinical CIP-resistant isolates also appear only in addition to target-directed mutations, likely due to relatively higher fitness costs. This overall consistency with data from clinical isolates (with a notable exception of GyrA ParC variants of E. coli, as discussed above) is possibly driven by the relatively large bacterial population sizes maintained in the morbidostat setup, along with continuous competition at the level of growth rate (fitness) imposed by frequent dilutions. In contrast, more traditional experimental approaches are constrained by population-limiting bottlenecks, which cause minimal competition and contribute to the selection of low-fitness variants (23).

Overall, this study (as well as other similar studies [26–28, 74]) confirms that morbidostat-based experimental evolution provides a powerful approach to assess the dynamics and mechanisms of antimicrobial resistance acquisition in a broad range of pathogens. This methodology is scalable and applicable to known antibiotics as well as novel drug candidates. The utility of comparative resistomics to assess and triage drug candidates across a range of target pathogens is expected to manifest even in the early phase of antimicrobial drug development. Combined with standard efficacy and safety evaluation, such an assessment would contribute to the rational selection of compounds capable of providing lasting therapies in the field for longer periods of use.

## MATERIALS AND METHODS

### Morbidostat setup.

The experimental evolution of CIP resistance was performed using an optimized version of a morbidostat device, which was engineered and validated in our previous study on the evolution of triclosan resistance in E. coli (28). The general design is based on the principles described in references 26 and 27 extending the chemostat approach toward the evolution of drug resistance. In the morbidostat, culture densities are maintained by regular automated dilutions with media containing or not containing antibiotic. This leads to gradual adaptation of bacterial populations to higher drug concentrations.

The detailed technical description of morbidostat hardware and accompanying software is provided at GitHub (https://github.com/sleyn/morbidostat_construction). Briefly, the main components of the device (Fig. 1a) are (i) six 20-ml glass tubes used as bioreactors, with magnetic stir bars and cap assemblies containing three air-tight needle ports for *(a)* introduction of fresh media and continuous airflow for culture aeration, *(b)* liquid displacement after dilution, and *(c)* sample collection; (ii) silicone rings used to secure reactors in three-dimensionally printed plastic housings which each contain a laser and a sensor diode for measuring culture turbidity; (iii) a small air pump to provide aeration for growing cultures (fitted with 0.22-μm-pore-size filters to block contamination in air feedlines) and enable liquid displacement from reactors (over a fixed level corresponding to a total volume of 20 ml); (iv) a thermoregulated heater and fan to control the temperature inside the morbidostat enclosure; (v) two 2-liter bottles with tubing connecting each to a peristaltic pump that controls the flow of media (with and without antibiotic) to the reactors, where an assembly of 12 check valves (2 valves per reactor, each connected with one of the two pumps) controls delivery of media to individual reactors during each dilution; and (vi) a six-position magnetic stir plate which agitates cultures and enables mixing of media upon dilution. An Arduino-based microcontroller is programmed to control the following main parameters of the run: (i) enclosure temperature, (ii) time between dilutions (cycle time [CT]), and (iii) selection of the volume delivered by pump 1 (medium without drug) or pump 2 (medium with drug) for each culture dilution, depending on the culture turbidity and growth rate in each tube. A user interface for parameter manipulation and real-time status display (including growth curves) is run on a PC using MegunoLink software (https://www.megunolink.com/; v.1.17.17239.0827 for the CEC-2, CEC-4, and CAB runs and v.1.32.20005.0105 for the PAC-1 and PAC-2 runs).

Automated dilutions are controlled using the following encoded logic (Fig. 1b).

1. In the active “dilution mode,” the optical density at the end of the current cycle (OD1) is compared with three parameters: (i) the predefined lower threshold (LT; typically ≤0.15), (ii) the predefined drug threshold (DT; typically ∼0.3), and (iii) the OD reached during the previous cycle (OD0). All OD values are corrected by adding the OD of fresh medium with antifoam.
2. The dilutions are always made by a predefined volume (V; typically 2 or 4 ml); if OD1 is equal to or above the DT and OD1 minus OD0 is above or equal to 0, the drug-containing medium (pump 2) is used with the interval corresponding to the predefined CT (typically 15 to 20 min), or else drug-free medium (pump 1) is added.
3. If OD1 is less than the LT, the system performs hourly dilutions with drug-free medium. In the beginning of the run, this allows all six cultures to reach the same minimal density (OD1 = LT) prior to entering the active dilution mode. During the run, this “safe mode” prevents a complete washout of the culture after an excessive dose of drug.

### Morbidostat runs.

Drug-free base medium consisted of cation-adjusted Mueller-Hinton broth (MHB) (Teknova) with a final concentration of 2% dimethyl sulfoxide (DMSO) (except in the CEC-2 and PAC-1 runs, which did not have DMSO) and a 1/2,500 dilution of antifoam SE-15 (Sigma). An autoclaved 1/50 concentration stock of the antifoam was added aseptically to the filter-sterilized (0.22 μm) MHB-DMSO mixture. The only difference between the base medium and the drug-containing medium was the addition of filter-sterilized ciprofloxacin (Sigma-Aldrich). Starting populations of the E. coli BW25113, A. baumannii ATCC 17978, and P. aeruginosa ATCC 27853 strains began from original glycerol stocks of parent organisms from the ATCC; stocks were streaked onto LB agar plates for isolation and grown overnight in MHB at 37°C. Liquid cultures were inoculated from single colonies and grown at 37°C, with shaking to an OD_600_ of 0.2 to 0.4, and then diluted to an OD_600_ of 0.02 with drug-free medium in reactors to begin the run. All reactors in a single evolutionary run with E. coli were started from a liquid culture sourced from one isolated colony, which was used to prepare a single glycerol stock for all studies. Different individual colonies were chosen to seed reactor cultures for A. baumannii and P. aeruginosa. Glycerol stocks from these starting cultures were preserved, and cell pellets were used to determine genomic sequences to account for the anticipated larger diversity of preexisting variants (see below). Five separate evolutionary runs were performed: two with E. coli BW25113 (CEC-2 and CEC-4), one with A. baumannii ATCC 17978 (CAB-1), and two with P. aeruginosa ATCC 27853 (PAC-1 and PAC-2). Of the two E. coli runs, CEC-2 was performed under a steeper increase in drug concentration than CEC-4. This was achieved by using a higher drug concentration in pump 2 medium, 0.156 mg/liter or 10-fold the CIP MIC value (0.0156 mg/liter as determined for the unevolved strain in the same medium), which was used in the beginning of the run, increasing to 0.468 mg/liter (30 times the MIC) on day 2 of evolution and then to 2.34 mg/liter (150 times the MIC) on day 4. The second E. coli run (CEC-4) used a CIP gradient starting from the lower concentration of 0.001 mg/liter (0.625 times the MIC) and gradually increasing up to 1.248 mg/liter (80 times the MIC) over a more extended time period (6 days). The CAB-1 run started at 0.195 mg/liter (1.25 times the MIC, given a MIC equal to 0.156 mg/liter for the A. baumannii ATCC 17978 unevolved strain) and progressed up to 40 times the MIC in 4 days. For morbidostat runs with P. aeruginosa, a modified control software was used (https://github.com/sleyn/morbidostat_v2_construction) which allows the user to vary the volume of drug-containing medium added at every dilution step. Therefore, the medium in pump 2 remained at the concentration of 7.8 mg/liter (25 to 50 times the MIC, as for the MIC of 0.156 to 0.313 mg/liter for the unevolved P. aeruginosa ATCC 27853 strain) throughout the experiment in both the PAC-1 and PAC-2 runs, with a continual increase in drug concentration controlled through the software alone.

Over the course of all runs, dilutions were performed with a volume of 4 ml (20% of the reactor volume). The calculated changes in drug concentration in each reactor were plotted (as in Fig. 1c to e) along with recorded changes in OD (see Text S1 and Fig. S2 for a complete set of parameters and plots generated for each run). As a result of optimization of the originally published morbidostat device and workflow (28), all runs were performed continuously (up to 6 days) without loss of sterility in bottles and feedlines. In the case of P. aeruginosa (but not E. coli or A. baumannii), the process included daily transfer of the culture (along with sample collection) to a fresh sterile glass reactor tube in order to minimize laser interference from biofilm gradually accumulating on the walls of the reactor. Otherwise, 10-ml samples were collected with a fresh sterile syringe via a dedicated needle port at one (or two) time points each day based on the OD, growth rates, and drug concentrations established in each reactor. All collected samples were used to prepare glycerol stocks (for further clonal analysis) and to extract genomic DNA from frozen cell pellets (prepared from the main portion of each sample).

10.1128/mBio.00987-21.1 TEXT S1 Detailed description of morbidostat experimental conditions for each morbidostat run (I) and the sequencing data analysis pipeline (II). Download Text S1, PDF file, 0.3 MB. Copyright © 2021 Zlamal et al. 2021 Zlamal et al. https://creativecommons.org/licenses/by/4.0/ This content is distributed under the terms of the Creative Commons Attribution 4.0 International license.

### Genomic DNA extraction and sequencing.

DNA was extracted using GenElute bacterial genomic DNA kit protocol NA2110 (Sigma-Aldrich) according to the protocol for Gram-negative cells. Total DNA from evolutionary-run samples were extracted from frozen cell pellets. DNA of selected clones was extracted from fresh pellets of liquid cultures grown 8 to 16 h at 37°C.

Nonamplified DNA libraries for Illumina sequencing of all population samples (and some of the analyzed clones) were prepared using a NEBNext Ultra II FS DNA library prep kit for Illumina modules E7810L and E7595L (New England BioLabs) by following the manufacturer’s protocol for use with inputs of ≥100 ng, with modifications to eliminate PCR amplification steps. IDT for Illumina TruSeq DNA UD index 20022370 or TruSeq DNA CD index 20015949 (Integrated DNA Technologies, Illumina) was used in place of NEBNext adaptors. Library size selection and cleanup were performed using AMPure XP beads (Beckman Coulter) according to the NEB protocol. An alternative faster and more cost-efficient approach was used for up to 96-plex sequencing of DNA from some of the analyzed clones. Clone DNA was prepared for Illumina sequencing using the plexWell PW384 kit and included adaptors (seqWell) by following manufacturer instructions.

Prepared libraries were quantified using a NEBNext Library Quant kit for Illumina E7630L (New England BioLabs) and pooled with volumes adjusted to normalize concentrations and provide for ∼1,000-fold genomic coverage for population samples (20 to 30 samples per HiSeq lane, depending on the genome size) or ∼200-fold coverage (up to 96 samples per lane) for clones. Library size and quality were analyzed with the 2100 Bioanalyzer instrument (Agilent). Pooled DNA libraries were sequenced by Novogene Co. on an Illumina HiSeq X10 or HiSeq 4000 machine using a paired-end 150-bp read length.

Nanopore long-read sequencing was used to verify and complete assemblies for unevolved genomes. DNA samples were prepared for long-read Nanopore sequencing using the Nanopore SQK-RBK004 rapid barcoding guide DNA (gDNA) sequencing kit (Oxford Nanopore Technologies) according to the manufacturer protocol and sequenced using MinION and flow cell FLO-MIN106.

### Sequence data analysis and variant calling.

The depiction of the sequencing data analysis pipeline (Fig. S3) and statistics for each sample (coverage, number of reads, and percentage of mapped reads) are provided in the supplemental material (Data Set S1). Briefly, Illumina sequencing read quality was assessed with FastQC v.0.11.8 (https://www.bioinformatics.babraham.ac.uk/projects/fastqc/). Adaptor trimming was performed using Trimmomatic v.0.36 (75). In population sequencing data analysis, we used BWA MEM v.0.7.13 for read alignment against reference genomes (76). SAM and BAM file manipulations were performed with Picard v.2.2.1 tools (https://broadinstitute.github.io/picard/) and SAMtools v.1.3 (76). Realignment and base quality score recalibration were performed with the LoFreq Viterbi module (77) and Genome Analysis Toolkit v.3.5 (78). For variant calling, SNPs and small indels were identified with LoFreq v.2.1.3.1 (77). IS element rearrangements in population samples were predicted by a new ijump software developed for this purpose (https://github.com/sleyn/ijump). IS elements in reference genomes were predicted using the ISfinder database (79). Variant effects were annotated with SnpEff v.4.3 (80). VCF file manipulations were performed with bcftools v.1.3 (81). Clonal sequencing data were analyzed using breseq software (82). Copy number variations were checked using the CNOGpro v.1.1 R language package (83). R v.3.6.0 was used. Repeat regions were masked based on analysis produced by MUMmer v.3.1 (84).

10.1128/mBio.00987-21.4 FIG S3 Computational pipeline for a primary analysis of population sequencing data (data shown in black hexagons). Processes and software are shown in rectangles and rounded rectangles, respectively. Frames indicate aims of the parts of the analysis. Download FIG S3, PDF file, 0.1 MB. Copyright © 2021 Zlamal et al. 2021 Zlamal et al. https://creativecommons.org/licenses/by/4.0/ This content is distributed under the terms of the Creative Commons Attribution 4.0 International license.

A *de novo* assembly of the Acinetobacter baumannii ATCC 17978 reference genomes for each of the six clones (samples A1 to A6) used as the starting point for experimental evolution was accomplished by a hybrid approach combining Illumina (short-read) and Nanopore (long-read) data. Nanopore reads were base-called and demultiplexed using Albacore v.2.3.4 base-caller (available on the ONT community site). To increase coverage with long reads, we performed a second round of demultiplexing of unclassified reads with Porechop v.0.2.4 (https://github.com/rrwick/Porechop). Adaptors were trimmed with Porechop. Both reads called with Albacore and Porechop were combined and used along with Illumina reads to make *de novo* assembly with SPAdes v.3.13.0 (85). The assembly was annotated using the RASTtk Web server (86).

For detailed description of sequencing data processing, see Text S1.

### MIC determination.

MIC values were determined for parent strains and selected individual clones to connect mutations and genome rearrangements with resistance phenotype. MIC assays were prepared with 2-fold increasing concentrations of ciprofloxacin by the broth dilution method by following the CLSI and EUCAST standard protocols, using resuspended fresh colonies in cation-adjusted Mueller-Hinton broth medium (87). Measurements were performed using (i) a growth curve method with microtiter plates at a wavelength of 600 nm in a BioTek ELx808 plate reader at 37°C (for E. coli and A. baumannii) or (ii) an endpoint analysis (for P. aeruginosa, results were read at 17 h).

### Data availability.

Clonal and population sequencing data are available in the SRA database under BioProject accession number PRJNA598012 ; the novel A. baumannii ATCC 17978 annotated assembly is available in the European Nucleotide Archive under sample accession number ERS4228590. The reference genomes were downloaded from the PATRIC database (88). PATRIC IDs are 679895.18 for E. coli BW25113 and 287.6323 for P. aeruginosa ATCC 27853. Custom code and a detailed description of sequencing data processing was deposited in GitHub (https://github.com/sleyn/paper_cipro_EC_AB_PA_2021).
