# Supplementary material for: Shared and Unique Evolutionary Trajectories to Ciprofloxacin Resistance in Gram-Negative Bacterial Pathogens
Source: mBio. 2021 Jun 22;12(3):e00987-21. doi: 10.1128/mBio.00987-21 (PMC8262867; doi:10.1128/mBio.00987-21)
Supplement: FIG S2 [file mbio.00987-21-sf002.pdf]

Supplementary Figure S2. OD profiles (black line) and calculated drug concentration profiles (red line) obtained in the course of experimental evolution of ciprofloxacin resistance in (**A**) *Escherichia coli* BW25113, CEC-2 run; (**B**) *Escherichia coli* BW25113, CEC-4 run; (**C**) *Acinetobacter baumannii* ATCC17978, CAB-1 run; (**D**) *Pseudomonas aeruginosa* ATCC27853, PAC-1 run; (**E**) *Pseudomonas aeruginosa* ATCC27853, PAC-2 run. The right axis shows CIP concentration (xMIC) as fold-change relative to MIC value of respective unevolved strains. Sequenced samples marked with arrows.

A

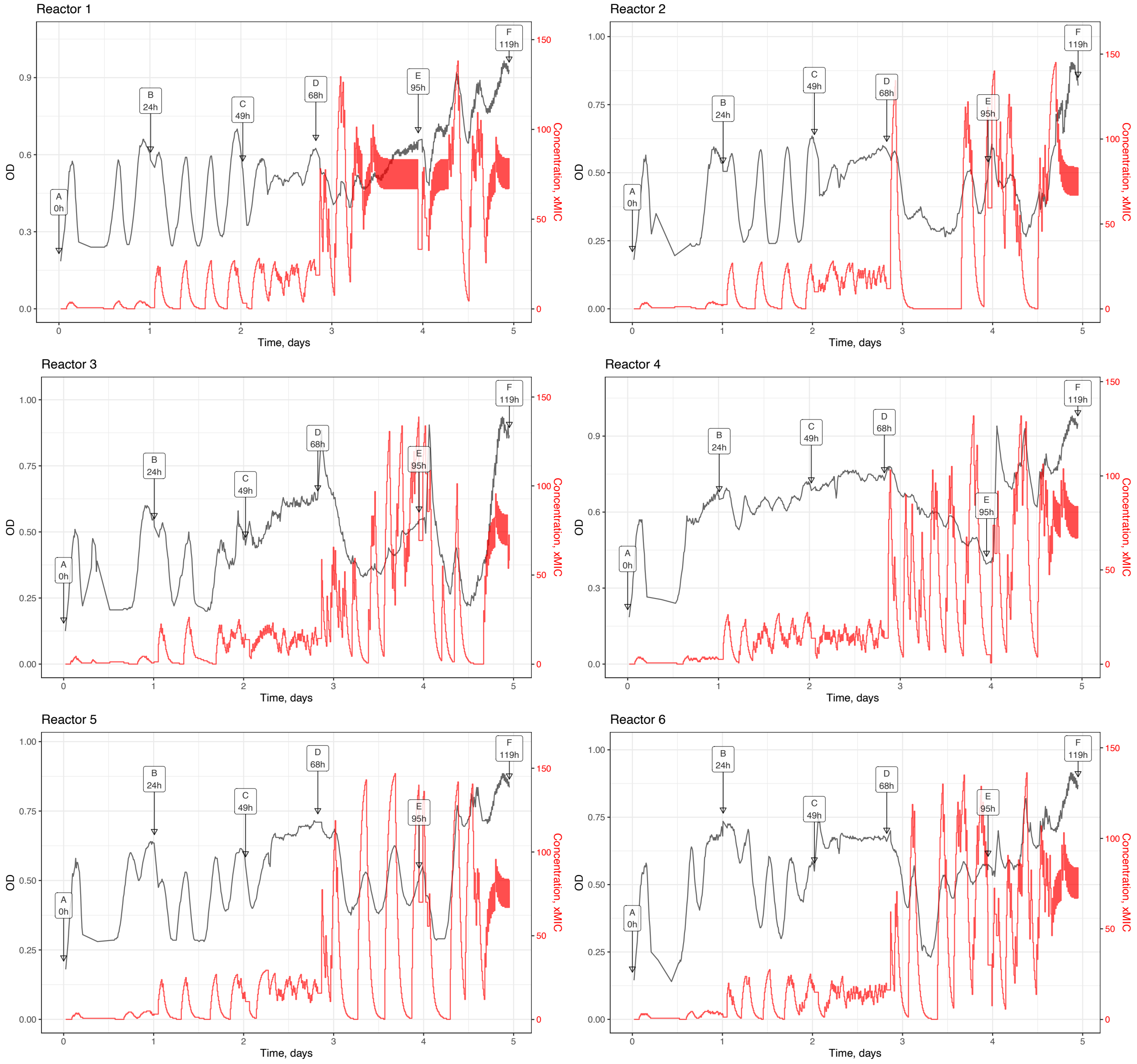

B

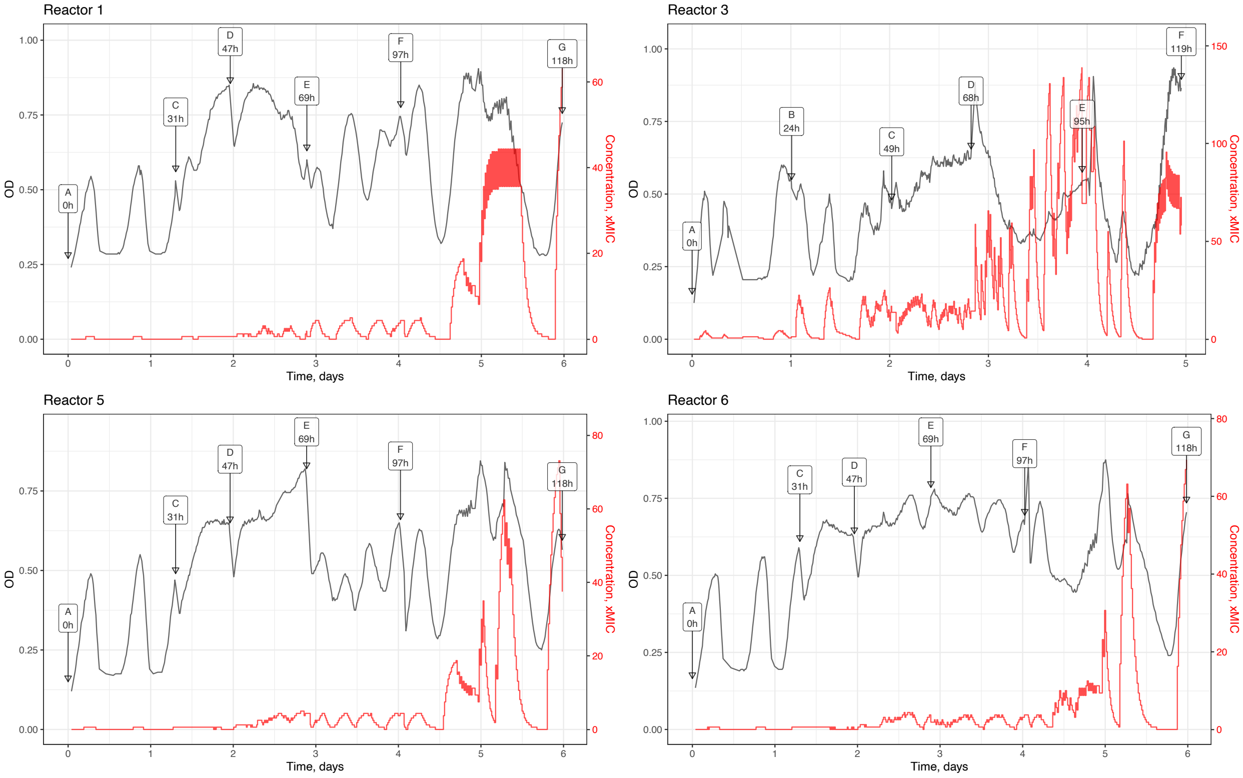
Reactor 1
Reactor 3
Reactor 5
Reactor 6
A
0h
B
24h
C
31h
C
49h
D
47h
D
68h
E
69h
E
95h
F
97h
F
119h
G
118h
OD
Concentration, xMIC
Time, days

C

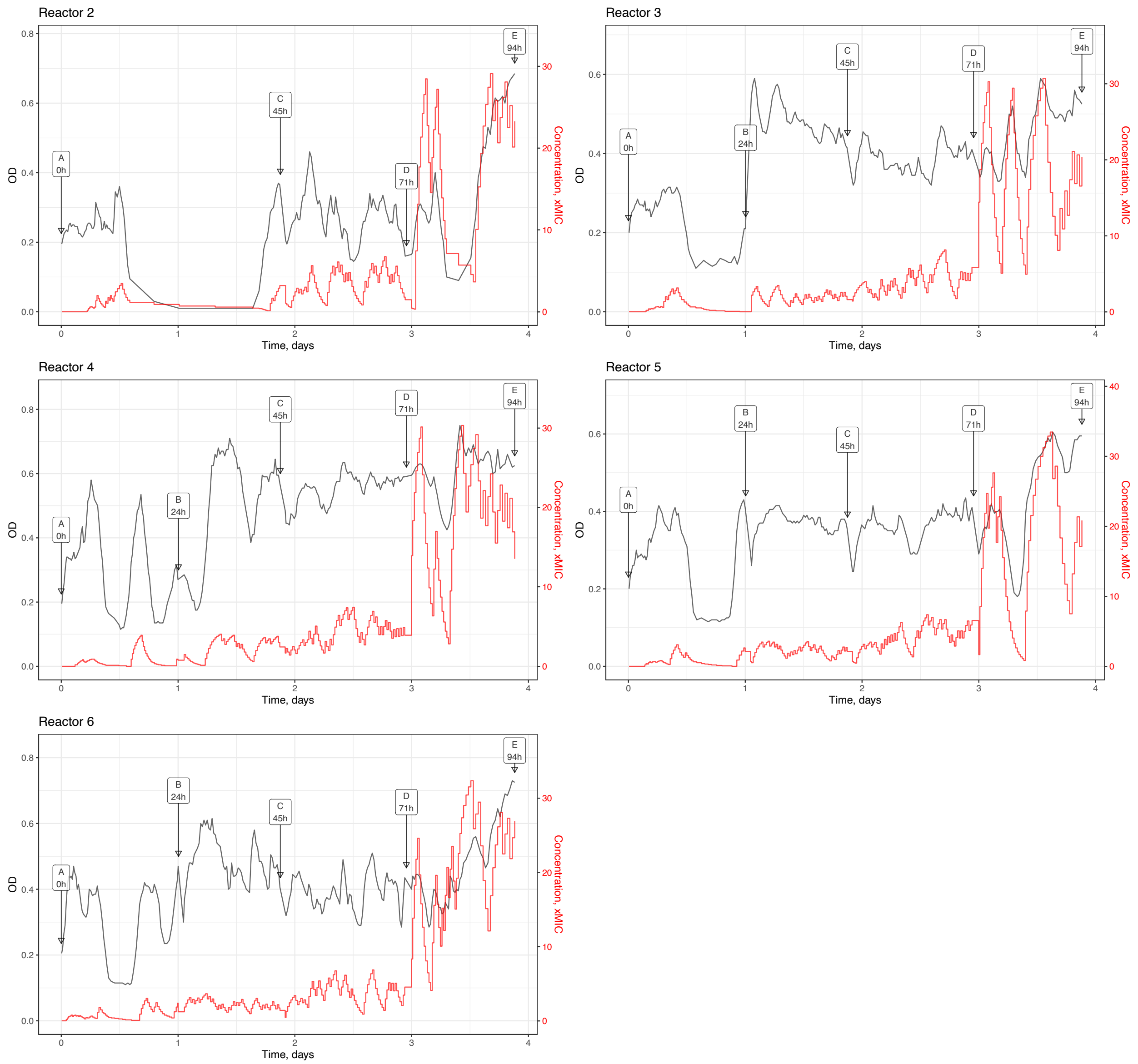
Reactor 2
Reactor 3
Reactor 4
Reactor 5
Reactor 6
A
0h
B
24h
C
45h
D
71h
E
94h
OD
Concentration, xMIC
Time, days
0.0
0.2
0.4
0.6
0.8
0
10
20
30
40
0
1
2
3
4

D

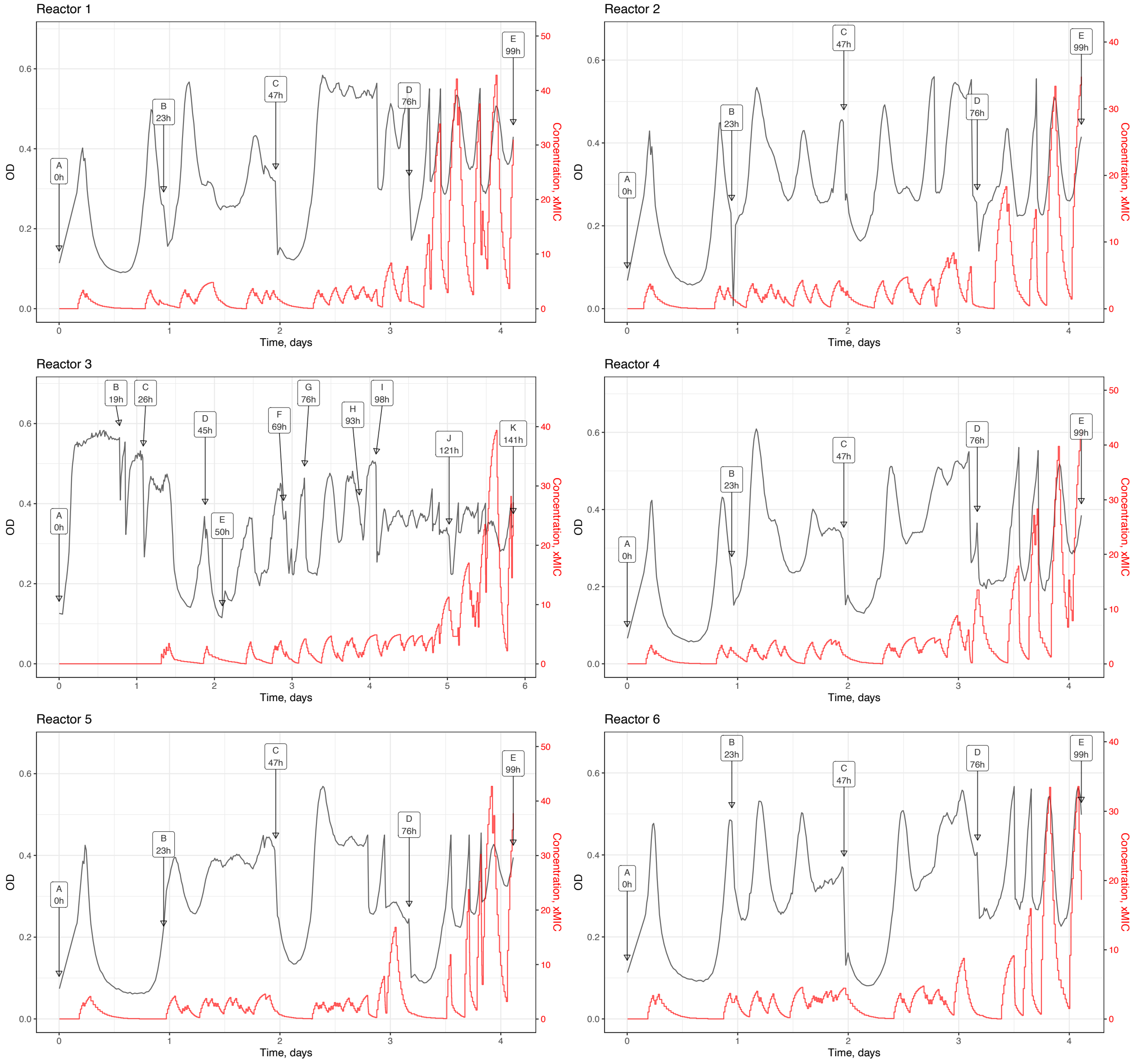

Reactor 1
Reactor 2
Reactor 3
Reactor 4
Reactor 5
Reactor 6
OD
Concentration, xMIC
Time, days
A 0h
B 23h
C 47h
D 76h
E 99h
B 19h
C 26h
D 45h
E 50h
F 69h
G 76h
H 93h
I 98h
J 121h
K 141h

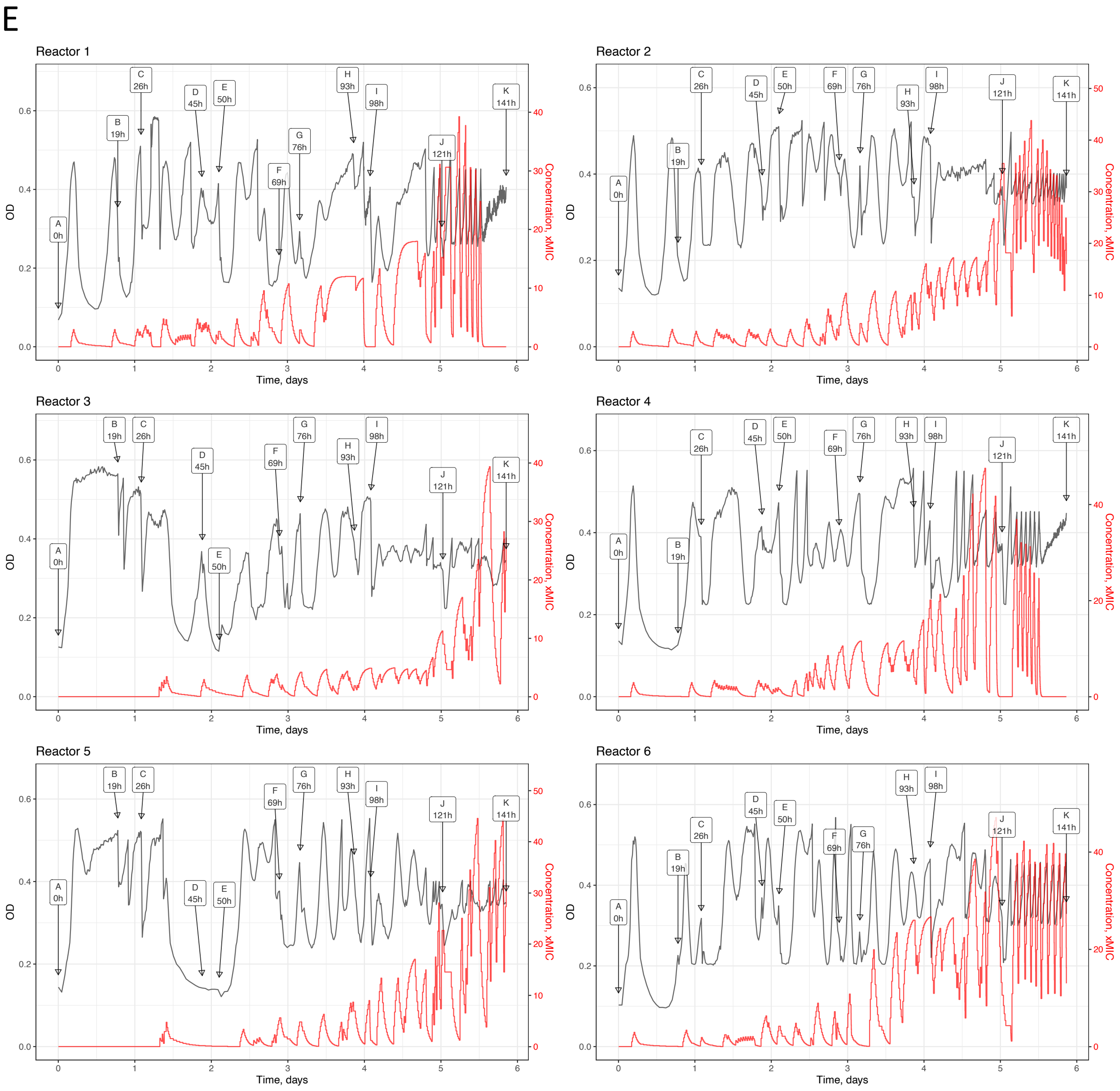
E
Reactor 1
Reactor 2
Reactor 3
Reactor 4
Reactor 5
Reactor 6
OD
Concentration, xMIC
Time, days
A 0h
B 19h
C 26h
D 45h
E 50h
F 69h
G 76h
H 93h
I 98h
J 121h
K 141h
