# Supplementary material for: Shared and Unique Evolutionary Trajectories to Ciprofloxacin Resistance in Gram-Negative Bacterial Pathogens
Source: mBio. 2021 Jun 22;12(3):e00987-21. doi: 10.1128/mBio.00987-21 (PMC8262867; doi:10.1128/mBio.00987-21)

Supplementary Figure 3. Computational pipeline for a primary analysis of population sequencing data. Data shown in black hexagons. Processes and software shown in rectangles and rounded rectangles respectively. Frames indicate aims of the parts of the analysis.

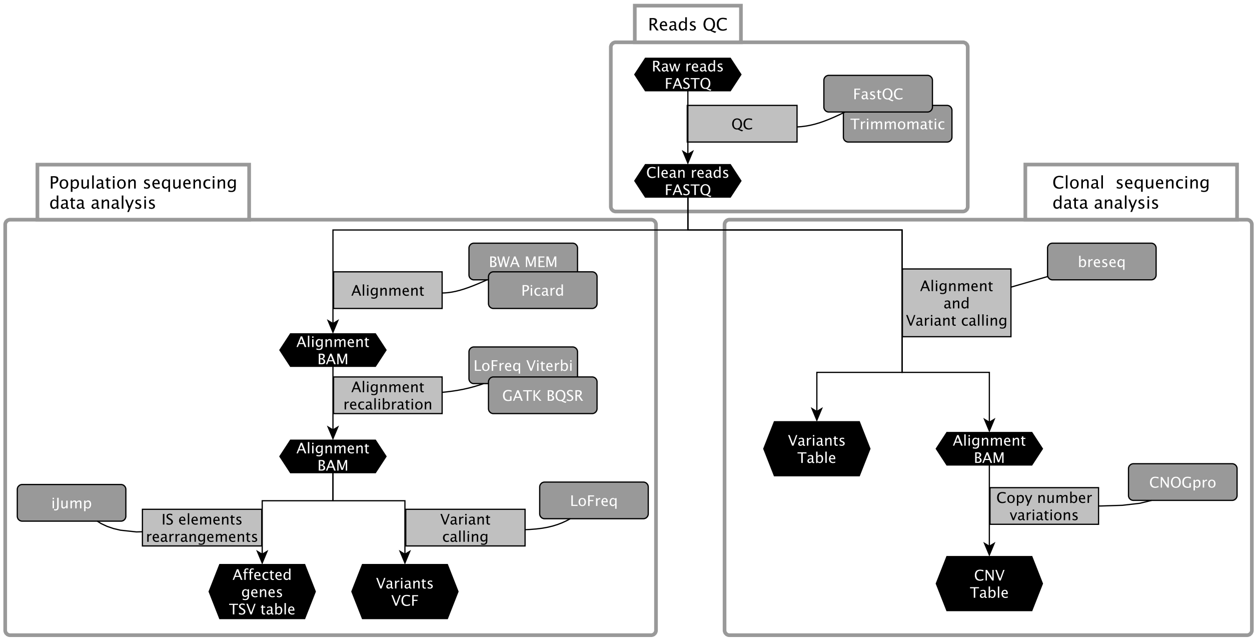

Supplement: FIG S3 [file mbio.00987-21-sf003.pdf]
